# Integrated geophysical assessment of the Abu Roash C member for hydrocarbon prospectivity in the Sitra Field Abu El Gharadig Basin, Egypt

**DOI:** 10.1038/s41598-025-11649-9

**Published:** 2025-07-24

**Authors:** Hadeel M. Kamel, Walid M. Mabrouk, Ahmed M. Metwally

**Affiliations:** Faculty of Science, Geophysics Department, Cairo University, Giza, 12613 Egypt

**Keywords:** Abu El-Gharadig Basin, Borehole image, Abu Roash C, North Western Desert, Core slabs, Geology, Geophysics

## Abstract

The Abu Roash C Member in Egypt’s Sitra Field (Abu El-Gharadig Basin, Western Desert) has been comprehensively assessed for its hydrocarbon reservoir potential through an integrated geological and geophysical study. The analysis combined well log data from four wells, core samples, high-resolution borehole images (captured via the Oil Mud Reservoir Imager – OMRI), and 2D post-stack time migration (PSTM) seismic interpretation. Petrophysical evaluation included log quality control, identification of hydrocarbon-bearing intervals, and quantification of key reservoir parameters (1) Shale volume (2) Effective porosity (3) Water saturation. Between wells Sitra-8–13 and Sitra-8–17, a laterally continuous sandstone unit was identified, demonstrating favorable petrophysical characteristics consistent with good reservoir quality with average porosity 11%, shale volume 9% and water saturation 56%. This reservoir is bounded by fine-grained siltstones and shales, interpreted as effective sealing units. Seismic interpretation revealed NW–SE oriented fault systems, indicating potential structural traps conducive to hydrocarbon entrapment. Lithological and petrographic analyses—encompassing core descriptions, lithofacies classification, and porosity measurements—suggest a paleoenvironment of shallow marine to marginal marine setting, likely influenced by tidal and storm processes, which explains the observed lithologic heterogeneity. These integrated findings affirm the Abu Roash C Member as a viable conventional hydrocarbon reservoir and provide critical insights for guiding future exploration and development in the basin

## Introduction

Fluid production can be significantly hindered by reservoir heterogeneity, which often arises from structural complexity, stratigraphic variations, and diagenetic modifications that disrupt the continuity of pore systems. Accurately identifying and analyzing these subsurface characteristics is crucial for efficient hydrocarbon exploration and production. Borehole imaging is a key technique that enables detailed examination and structural geological interpretation of the reservoir^1–5^. Additionally, core analysis remains indispensable in stratigraphic interpretation, involving the description, classification, and naming of sedimentary rocks based on stratal patterns. This approach enhances our understanding of reservoir characteristics and fluid distribution^6^.

The Western Desert of Egypt has recently emerged as one of the country’s most promising regions for oil and gas development. This surge in exploration success stems from a series of notable discoveries dating back to the 1980s^1,7–23^. One of the key hydrocarbon-bearing intervals in this region is the Abu Roash Formation, particularly the Abu Roash C Member within the Abu El-Gharadig Basin, which has shown evidence of sandstone streaks. These features highlight the potential for further hydrocarbon accumulations and underline the need for detailed investigation^24^.

**Fig. 1:**
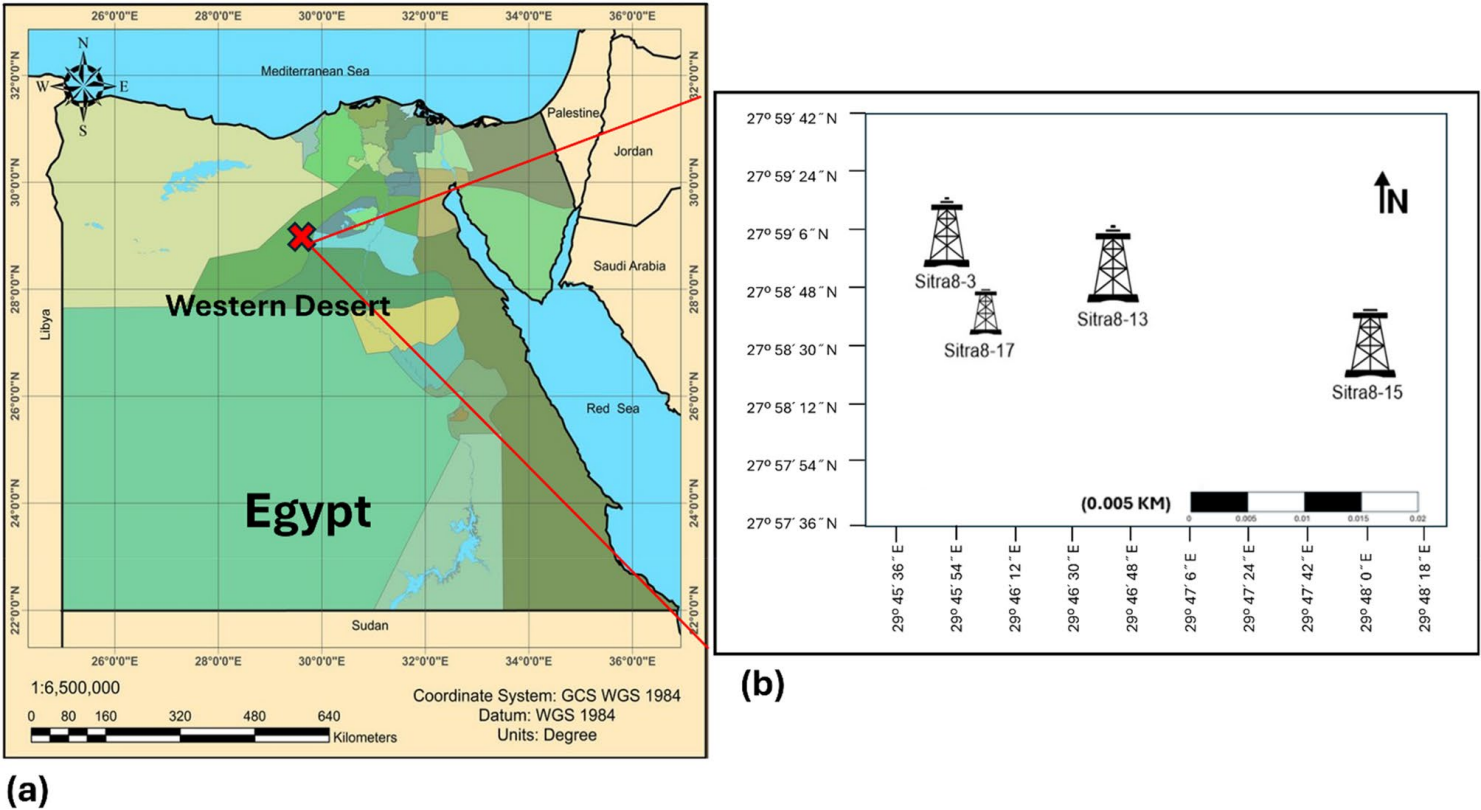
(**a**) Location map of the research region (**b**) Location map depicting the four analyzed wells in the Sitra Field.

This study aims to provide a detailed interpretation of the Abu Roash C Member within the Sitra Field of the Abu El-Gharadig Basin, located in Egypt’s Western Desert (Fig. 1). The analysis utilizes borehole image logs, core sample evaluation, and enhances previous methodologies by integrating petrophysical data and seismic interpretation to achieve more robust and reliable results. The Sitra Field is situated in the north Western Desert, specifically in the central-western portion of the Abu El-Gharadig Basin. It lies within the lease area operated by the Badr El-Din Petroleum Company (BAPETCO), covering a total area of 322.4 km^2^ ref.^21,22^. The region contains several structural closures, with significant production occurring in the Sitra 8 section, which is the focus of this study. This field is positioned on the downthrown side of the Basin’s major border fault. The coordinates of the study area are between latitudes 29° 44′ 41′′ and 29° 48′ 00′′ N, and longitudes 27° 56′ 27′′ and 28° 00′ 00′′ E, encompassing approximately 35 km^2^ ref.^1,3,4,22,25–27^ (Ibrahim Salema, 2015).

Given the presence of sandstone streaks in the Abu Roash C Member, this formation warrants a more refined and integrated geological and geophysical evaluation. Therefore, this research incorporates data from borehole images, open-hole logs, seismic interpretation, and core slabs from four wells. This multi-disciplinary approach is designed to assess the hydrocarbon potential of the Abu Roash C Member and to provide a more accurate structural and stratigraphic framework for exploration and development in the Sitra Field.

## Geologic settings

**Fig. 2:**
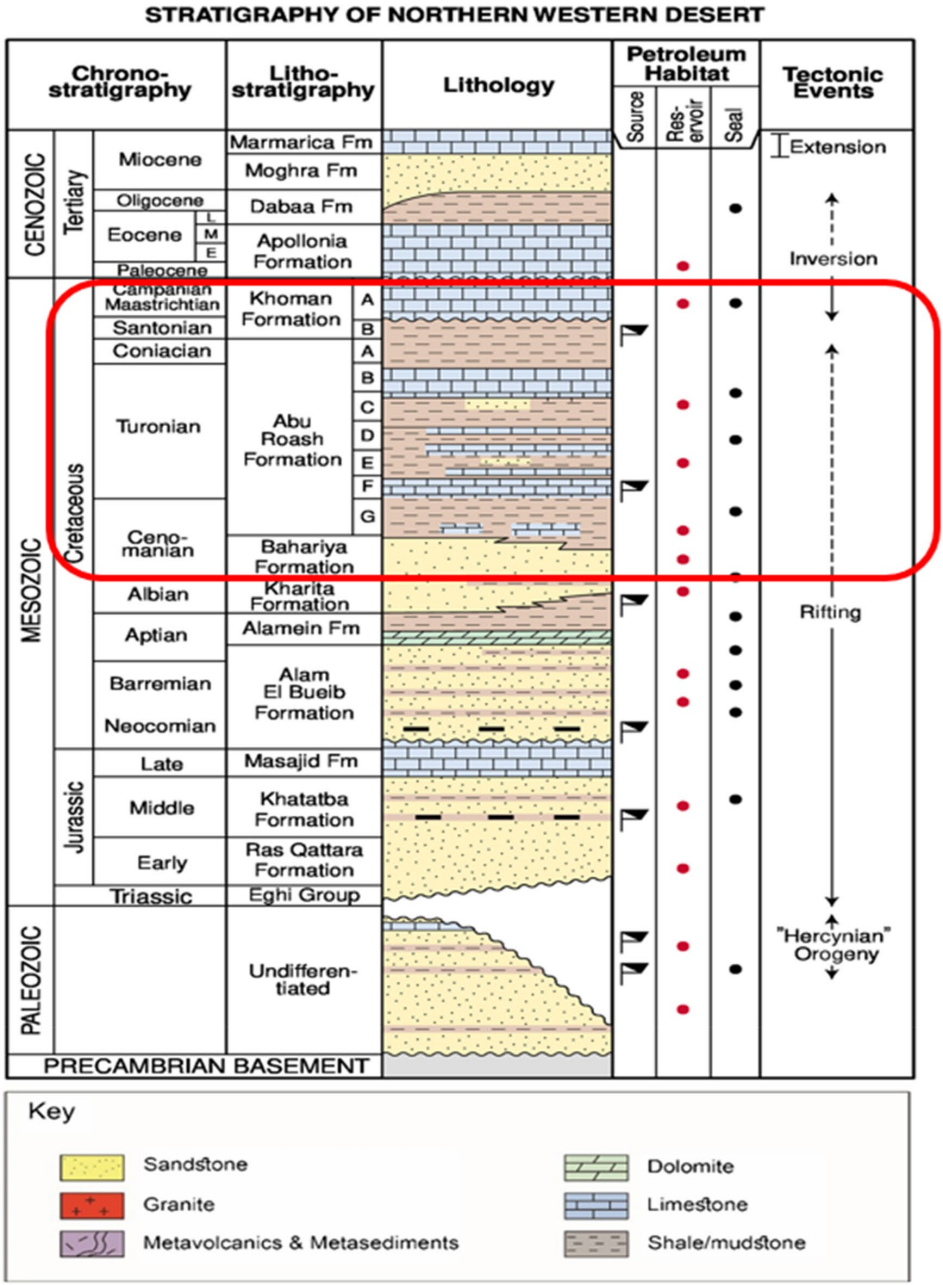
Stratigraphic column of northern Western Desert highlighting the Upper Cretaceous units modified after^25,30,34^.

Abu EL-Gharadig Basin is an intracratonic East–West trending rift Basin that may be found in the northern Western Desert of Egypt. The Abu El-Gharadig Basin spans from the Qattara Depression in the west to the Kattaniya horst in the east, surrounded by two basement uplifts, one to the north known as the Sharib-Sheiba ridge, and one to the south called Sitra uplift (Fig. 1) ref.^1,3,4,17,21,28–30^. Beginning in the Jurassic and continuing into the Early Cretaceous and continuing into Late Cretaceous, the rifting phase was the dominant phase. Alpine orogeny, which continued from the Late Cretaceous to the Tertiary era, is squeezed in a north-westerly direction during the Post-Rifting phase. This took place throughout the time period. The conclusion was reached that the northern Basin was significantly impacted by a compressional and shortening phenomenon. Because of this, the North-West-South East to East corridor was established. Southeast to the west, there were extensional structures that trended north-west and compressional structures that trended north-east-south-west, both of which led to an uplift phase and then eroded throughout the Santonian period. There were a number of structural traps that were generated as a consequence of the Basin inversion^17–20,28,31^. At some point in time during the late Early Cretaceous period, the Abu El-Gharadig Basin came into being. There were substantial periods of rising sea levels (transgressive) and floods that occurred throughout the post-rift stage of the Late Cretaceous period. These events led to the deposition of sedimentary rock members that were dominated by carbonate minerals^32^. Moreover, there were also shorter periods of dropping sea levels (regressive), during which lesser volumes of clastic material from land sources were deposited^3–5,25,27^. These findings were published in the three studies mentioned above. According to^17–20^, the Bahariya Formation, Abu Roash Formation, and Khoman Formation are the significant late Cretaceous geologic units that may be found in the northern Western Desert (Fig. 2). During the Middle Cenomanian epoch, the Bahariya Formation was produced, and the majority of its facies were composed of sandstone. Within the context of a broad and shallow sea shelf community. It was during the upper Cenomanian–Coniacian epoch that the Abu Roash Formation was deposited in a conformable manner on top of the Bahariya Formation. It reached its maximum thickness of around 3,200 feet at the depocenter of the Abu El-Gharadig Basin. It was subdivided into seven members, which were designated as A, B, C, D, E, F, and G. It went through maritime transgression and regression cycles simultaneously. There is an unconformity between the Khoman Formation and the Abu Roash Formation, which is located beneath the Khoman Formation from the Upper Cretaceous. As a consequence of this, the G, E, and C Members are mostly formed of fine siliciclastic sediments, whereas the F, D, B, and A Members are significantly more composed of carbonates. There is evidence that the Khoman Formation, which is composed of shale and limestone, was deposited in an outer shelf to a deep open marine environment^3,4,17–20,29,32,33^.

## Materials and methods

Four wells placed in the main producing closure of the Sitra 8 block (Sitra 8–3, Sitra 8–13,Sitra 8- 15, and Sitra 8–17) generated using QGIS 3.4 and SURFER 13 software presented in (Fig. 1) were supplied by Badr EL-Din company for the purpose of this study. Borehole image data for Abu Roash-C in wells (Sitra8-13 & Sitra8-17) were retrieved using the Oil Mud Reservoir Imager (OMRI) Tool that gives detailed, accurate pictures of the reservoir that answer geological and petrophysical questions. It is a high-resolution digital image tool of the wellbore down to one inch of vertical resolution, instead of one foot of vertical resolution, available with conventional logging tools. Moreover, core analysis for several Slabbed conventional Cores is performed to get an appropriate stratigraphic interpretation of the Abu Roash C late Cretaceous Member (Fig. 2). (Table 1) The core intervals belong to wells (Sitra 8–13 and Sitra 8–17). Adding to the previous, 2D seismic sections passing by wells (Sitra 8–3, Sitra 8–13, Sitra8-15, and Sitra 8–17) for better interpretation, and open-hole log data, including Gamma Ray, Density, Neutron, Sonic, and Resistivity, were analyzed using Techlog.2015..

**Table 1:** Shows the core interval in the two wells.

Well name	Core interval	Member
SITRA8-13	(2867.4 -2899)m	Abu Roash C
SITRA8-17	(2837.20- 2864.21)m	Abu Roash C

### Petrophysical analysis

This analysis aimed to identifying the zone of interest in Abu Roash-C by analyzing the petrophysical parameters of four wells, using data obtained from well logs (gamma ray log(GR), resistivity log, neutron-density log and sonic log).

Petrophysical analysis includes the following.

#### Log quality control

The process includes multiple steps and techniques aimed at detecting and resolving any anomalies or spikes in the acquired log data^13,21,23,35–37^.

#### Zone of interest and lithology identification

The identification of zones of interest relies on the enhancement of the deep resistivity log, which signifies hydrocarbon presence. Additionally, these zones should display lower gamma ray responses and exhibit density-neutron log crossovers, indicating porosity. The key parameters for lithology identification are derived from various log cross-plots, including density–neutron, sonic-neutron, M–N plot, and MID plot^13,21,23,35–37^.

#### Evaluation of key reservoir parameters:

Shale volume: to determine the shale content in a formation.

Effective porosity: the percentage of total pore space within a rock that allows fluid to flow throughout its entire volume.

Water saturation: to identify the hydrocarbon-bearing formation^13,21,23,35–37^.

### Core analysis

This study presents the sedimentological and petrographic interpretation results of conventional core samples, including lithofacies analysis entails a detailed lithologic description of the cores, breaking down the analyzed successions into their component lithofacies, relating the identified lithofacies suits into their most probable paleoenvironment in the cored successions.

### Borehole image

**Fig. 3:**
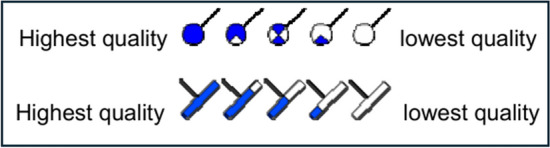
The five quality levels.

The Oil Mud Reservoir Imager (OMRI) Tool is a six-arm, each one with a 6-pair button electrical imaging tool that generates a color-coded resistivity image of the Formation approximately 0.5 inch (1.5 cm) behind the borehole wall. The borehole coverage is 57% in an 8.5-inch diameter wellbore, and the image resolution is 1 inch (25 mm). The processed OMRI image data over the logged intervals is good in quality. Almost all imaged sections display well-defined and interpreted bedding features and primary (depositional) and secondary (tectonic) structures^38^. Dip Classification: A total of 6 different planar features were defined in this study and used in enclosures and some figures. They are represented by various sets of colored symbols and tadpoles summarized in (Table 2). The pick quality can be identified by the fill inside the head of the tadpole and the base of the frac-pole. We use five quality levels and they are presented as follows (Fig. 3).

**Table 2:** Dip classification scheme.

Plane type	Symbol & color	Description
Shale/mudstone plane bedding/lamination		Mudstone and shale bedding/lamination with flat parallel nature or occasionally slightly undulated. Its attitude almost represents the post-depositional structural tilt
Sandstone flat bedding		Sandstone bedding; flat to low-angle dipping. Its attitude is sometimes used as a secondary indicator for post-depositional dip
Cross-bedding		Depositional-related inclined planes are commonly developed in sandstone, heterolitics, and occasionally in mudstone
Limestone flat bedding		Limestone bedding/lamination Its attitude is sometimes used as a secondary indicator for post-depositional dip
Heterolithics		Sandstone and mudstone interbedded lamination
Tectonic fracture		Resistive (anomaly light) natural plane being discordant to bedding without dislocation and juxtaposition of different lithologies
Fault		Resistive (anomaly light) natural plane being discordant to bedding with dislocation and juxtaposition of different lithologies

The automated measured true dip attitudes of all picked bedding and structural planes were presented in Rose diagrams and histograms. Such stereographic projections allow a ready visualization of planar spatial data distributions. They are used to define and calculate the mean dip of subpopulation clusters or to extract the axial trends from particular dip patterns. In addition to stereographic projections, a vector azimuth diagram showing azimuthally trends and changes of trends for specified groups of depositional bedding planes was also constructed. The construction starts at the bottom and then adds the dip directional component of each dip tadpole without any role of dip amounts. This type of plot is read from base to top and is useful in spotting dip trends and marking sharp excursions or breaks that signify geologic events as faults or unconformities^38,39^.

### Seismic interpretation

The evaluation was carried out on the post-stack time migration (PSTM) process of 2D seismic data to reduce structural uncertainty^40^ through the following:

#### Data preparation

Gather and process seismic data, ensuring quality control.

#### Synthetic seismogram

Simulate how seismic waves travel through different subsurface materials which involves gathering sonic and density logs from boreholes to calculate acoustic.

impedance and Reflection Coefficient at each boundary between layers of different seismic velocities, then we choose a seismic wavelet that represents the expected seismic wave shape as it travels through the subsurface and finally, convolve the reflection coefficients with the chosen wavelet to create the synthetic seismogram, which visually represents the expected seismic response.

#### Time-depth curve from check shot data

To measure the travel times of seismic waves to different depths and plot these measurements to create a depth-time function.

#### Well to seismic tie

Integration of well log data with seismic data. This process generally involves constructing synthetic seismograms from well log data and comparing them with seismic traces to ensure accurate geological interpretations.

#### Horizons picking and fault interpretation

Involves identifying reflection surfaces that represent geological features or boundaries to reflect an indirect image of the subsurface.

#### Two way time structural maps

TWT structural maps showing horizontal distance versus two-way travel time to represent the depth and geometry of these reflections, offering critical information about the subsurface characteristics.

#### Depth conversion

By using average velocity calculated from the T-D curve provided from the check shot data acquired in the available wells.

## Results and discussion

### SITRA8-13 well

#### Core analysis

**Fig. 4:**
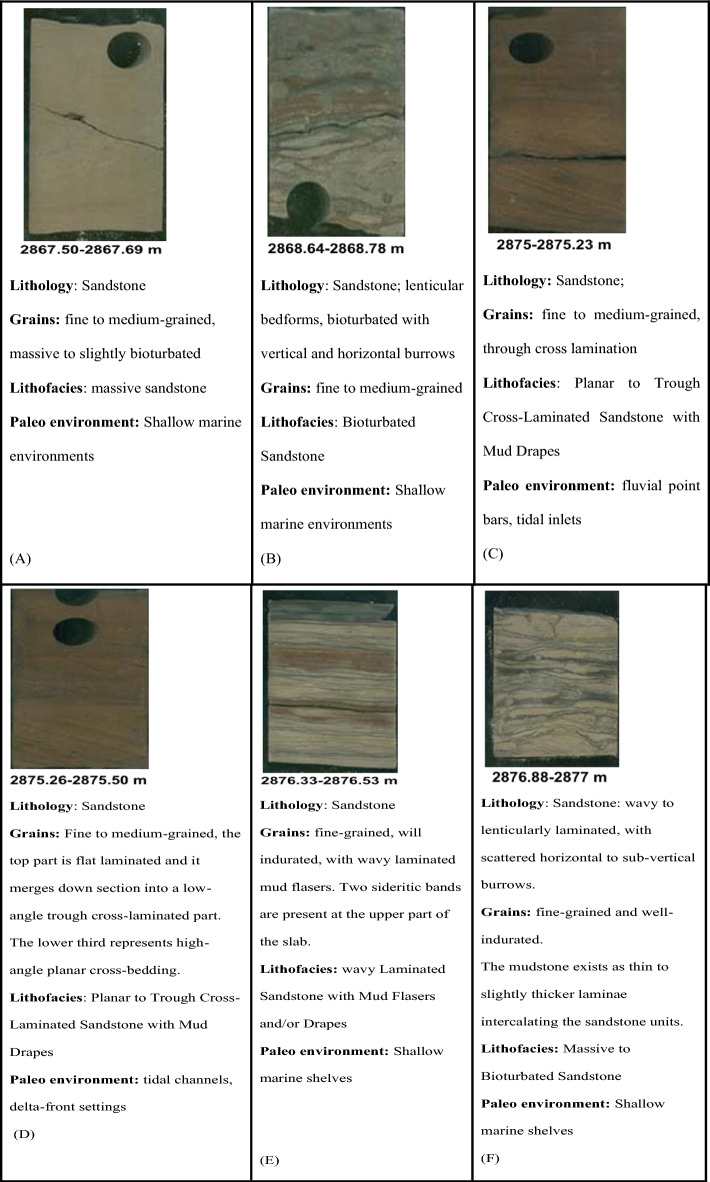

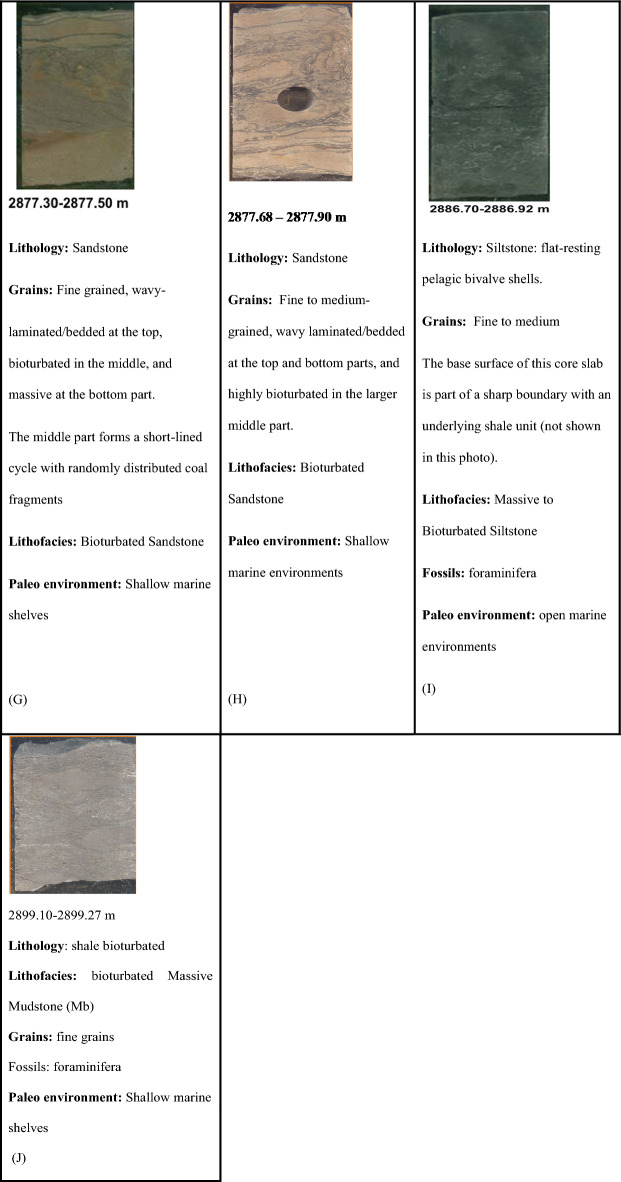
Different core slab intervals (**A**–**J**) in Abu Roash C, Turonian age through Sitra8-13 well.

Slabbed conventional Core in Sitra 8–13 well, measuring a total length of 36.7m (between measured depths of 2864.00m and 2900.70m). Figure 4 describes the lithology, sedimentary structure, and lithofacies classification of ten slab core samples in the late Cretaceous Turonian age Abu Roash C Member. The Presence of Coal Beds, the general upward-coarsening pattern of proper deltaic succession, the recurrent occurrence of mud drapes, variable degrees of bioturbation, and the presence of pelagic bivalves are all evidence for a tidally dominated deltaic depositional setting (Badr El-Din report, 2024 unpublished). Sitra8-13 well is primarily composed of sandstone interbedded with silt and shale. The sandstones (medium grained) possess good porosity, serve as reservoirs supporting hydrocarbon accumulation, while the shales function as seals.

The dominant sedimentary structures are bioturbated laminations and bedding, which influence permeability anisotropy by enhancing fluid flow through the creation of secondary porosity and affecting fluid migration pathways^22,41,42^.

The core interval indicates that the Abu Roash C in the Sitra8-13 well is composed primarily of sandstone. The sandstones are typically fine- to medium-grained, and their reservoir quality is influenced by Diagenetic processes like cementation and compaction, which reduce porosity, structural complexity, including faulting, which affects hydrocarbon migration and trapping and variable porosity and permeability, often due to facies changes and diagenetic overprints.

The depositional setting of the Abu Roash C Member reflects a shallow marine to coastal environment, likely influenced by transgressive–regressive cycles^26,40^.

#### Petrophysical analysis

**Fig. 5:**
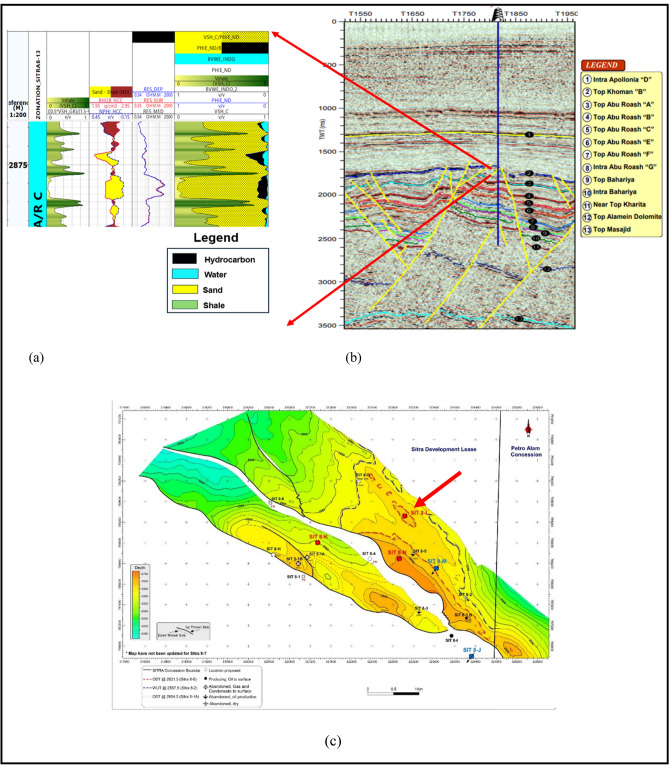
(**a**) Litho-saturation model showing the petrophysical characteristics while highlighting the zone of interest in Abu Roash C Member in Sitra8-13 (**b**) 2D interpreted seismic line through well SITRA8-13 (blue picked line represents top of Abu Roash C) (**c**) Depth structure map.

Petrophysical analysis was applied to determine the interval of interest in the Abu Roash C Member^21,43^, Fig. 5a shows the zone of interest which is represented by raising the deep resistivity log, which acts as a reliable signal for the existence of hydrocarbons. The area should also demonstrate decreased gamma-ray responses at the intersections of density-neutron logs. which signifies the existence of a zone that allows for the passage of substances and an average effective porosity of approximately 12%, Average shale volume 10% and 56% Water saturation (Table 3).

**Table 3:** Petrophysical analysis of Abu Roash C Member.

Well	Zones	Top	Bottom	Reference unit	Gross	Net	Av_Shale volume	Av_Porosity	Av_Water saturation
SITRA8-3	ABU ROASH C	2795 m	2879 m	M	84	54.312	0.206	0.094	0.704
SITRA8-13	ABU ROASH C	2822 m	2911 m	M	89	67.3	0.10	0.119	0.565
SITRA8-15	ABU ROASH C	2858 m	2875 m	M	17	6.992	0.172	0.063	0.703
SITRA8-17	ABU ROASH C	2792 m	2891 m	M	99	60.724	0.097	0.134	0.59

#### Borehole image

**Fig. 6:**
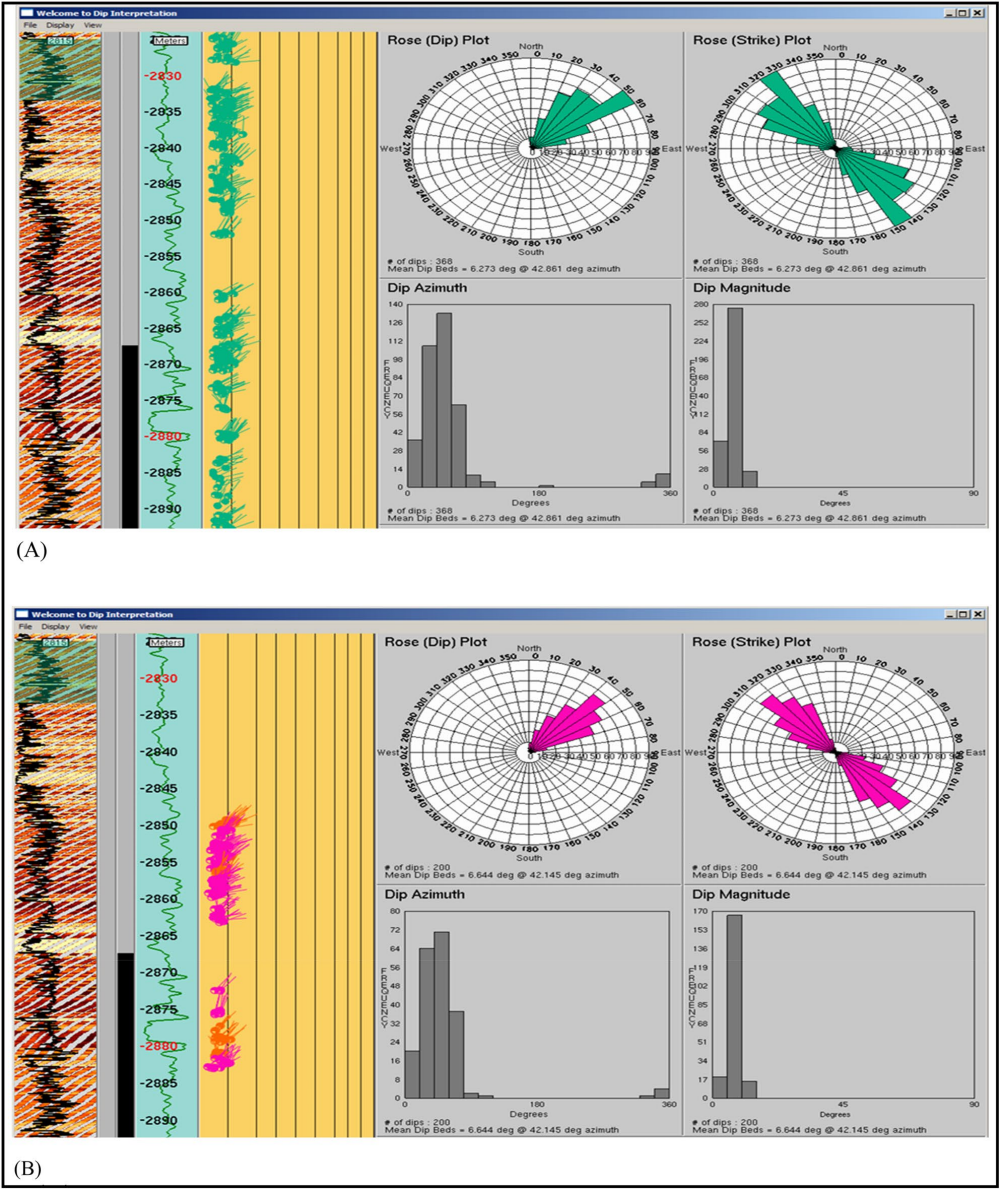
(**A**) Rose diagrams and histograms for (a) mudstone bed borders and lamination planes that were chosen from the Abu Roash C Member in Sitra8-13. When measured, their mean dip angle is 6°/N42°E, which means they hit mostly NW–SE and dip mostly NE. And for (**B**) a few sandstone (red) and heterolithics (magenta) bed boundaries picked from Abu Roash C Member in Sitra8-13. They strike generally NW–SE and dip NE with a calculated mean dip attitude measuring 7°/N42°E.

SITRA 8–13 well is nearly vertical with borehole conditions generally good all over the logged interval without a significant rugosity. In the borehole image, Abu Roash C Member was drilled in the measured depth interval from 2824 m down to 2891 m with a penetrated thickness of about 77 m. The studied interval of this Member consists mainly of mudstone interbedded with heterolithics and sandstone intervals (Table 2). Intrinsic features in the image revealed that (1) mudstone bed boundaries act as seal rock that can hydrocarbons (structural trap) and lamination planes generally strike NW–SE and dip mostly NE with a found average dip mood measuring 16,206 6°/N42°E as shown in Fig. 6A. (2) In Fig. 6B its few sandstone that act as reservoir & heterolithics bed boundaries and lamination planes that strike generally NW–SE and dip mostly due NE wich allow lateral hydrocarbon migration up toward fault trap with a calculated mean dip attitude measuring 7°/N42°E. The NW–SE strike could create a 3-way dip closure sealed by overlying mudstones. Both the mudstone and sandstone packages dipping NE at similar angles and directions suggest a layered, tilted structural panel. If there’s any faulting superimposed on this geometry like a fault trending NE–SW intersecting the dip direction this could develop localized closures ideal for hydrocarbon entrapment.

**Fig. 7:**
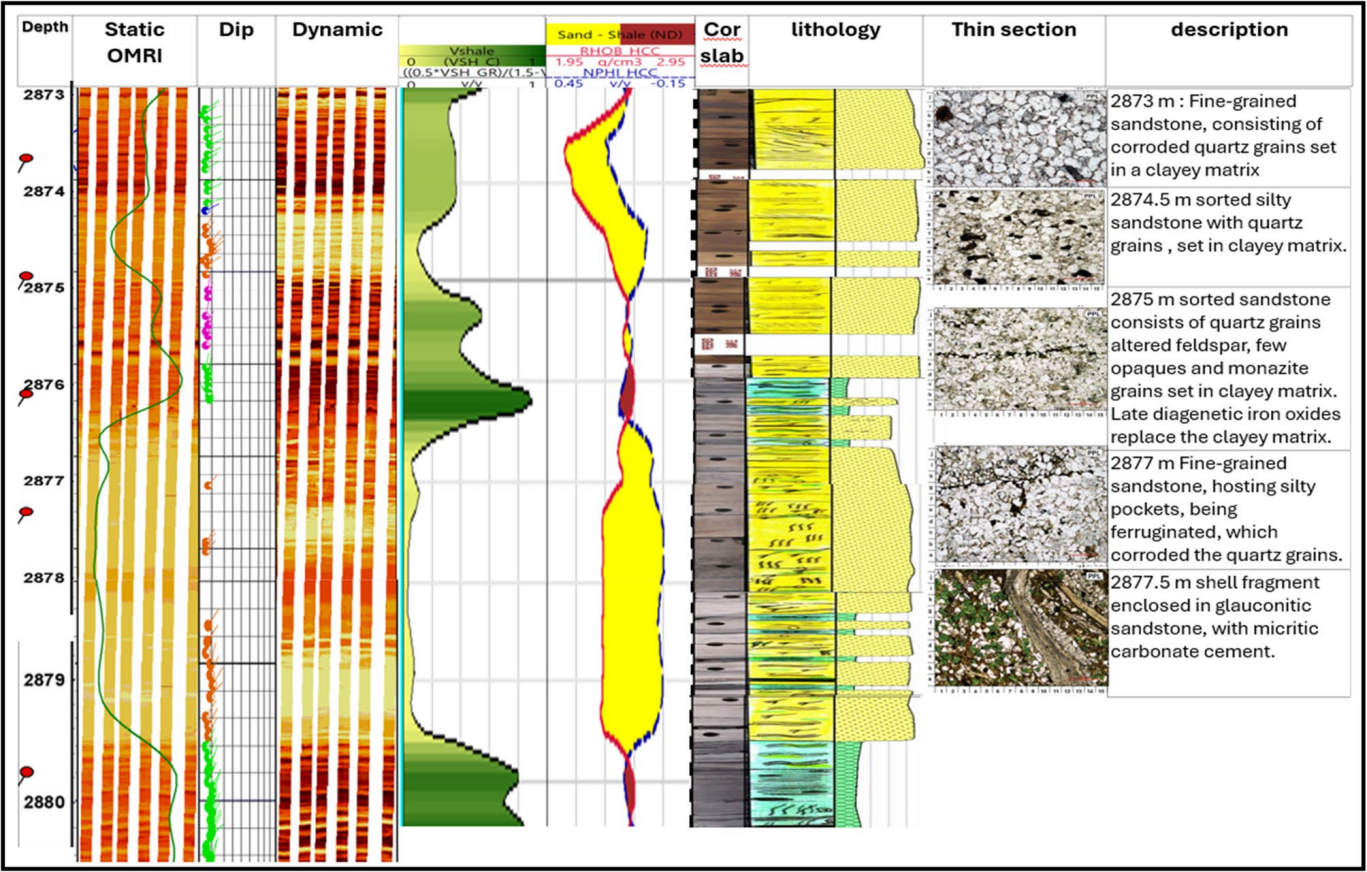
Integrating analysis of (borehole image, core and log data) in Abu Roash C Member Sitra8-13.

The integrated analysis of (borehole image, core, and log data) in Abu Roash C Member well sitra 8–13 showed in Fig. 7, and they confirmed each other. Borehole images reveal a sandstone interval that corresponds with low gamma-ray readings and a density-neutron crossover, correlating to the same core interval that composed of sandstone grains. A similar pattern was observed in^24,44^ research, supporting the present findings that the Abu Roash C reservoir is regarded as providing excellent reservoir quality because of its high porosity values.

#### Seismic interpretation

The Sitra development zone is structurally defined by a series of elongated horsts and grabens trending predominantly in an east–west direction. Hydrocarbon accumulations in the area are primarily confined within the sandstone reservoirs of the Abu Roash Formation^22^. At the Abu Roash C level, the Sitra 8 closure is segmented by multiple northwest-southeast-trending faults, resulting in several discrete reservoir compartments, as illustrated in Fig. 5b,c.

Seismic interpretation (Fig. 5b) reveals a prominent horst block bounded by two NW–SE oriented normal faults, likely formed during the Cretaceous rifting phase. This structural configuration suggests the presence of a potential hydrocarbon trap. The Sitra8-13 well, drilled within this horst block, intersects the Abu Roash C Member, which is flanked by the overlying Abu Roash A and B Members—both of which act as impermeable seals (see Fig. 2). This juxtaposition supports the interpretation of Abu Roash A and B as effective cap rocks for the Abu Roash C reservoir.

Further structural analysis, based on the structural map by Sarhan^24^, identifies an asymmetrical anticline trending ENE–WSW and plunging northeastward. This fold is crosscut by numerous extensional faults, which reflect the complex structural deformation associated with compressional tectonic events.

### Sitra8-17 well

#### Core analysis

**Fig. 8:**
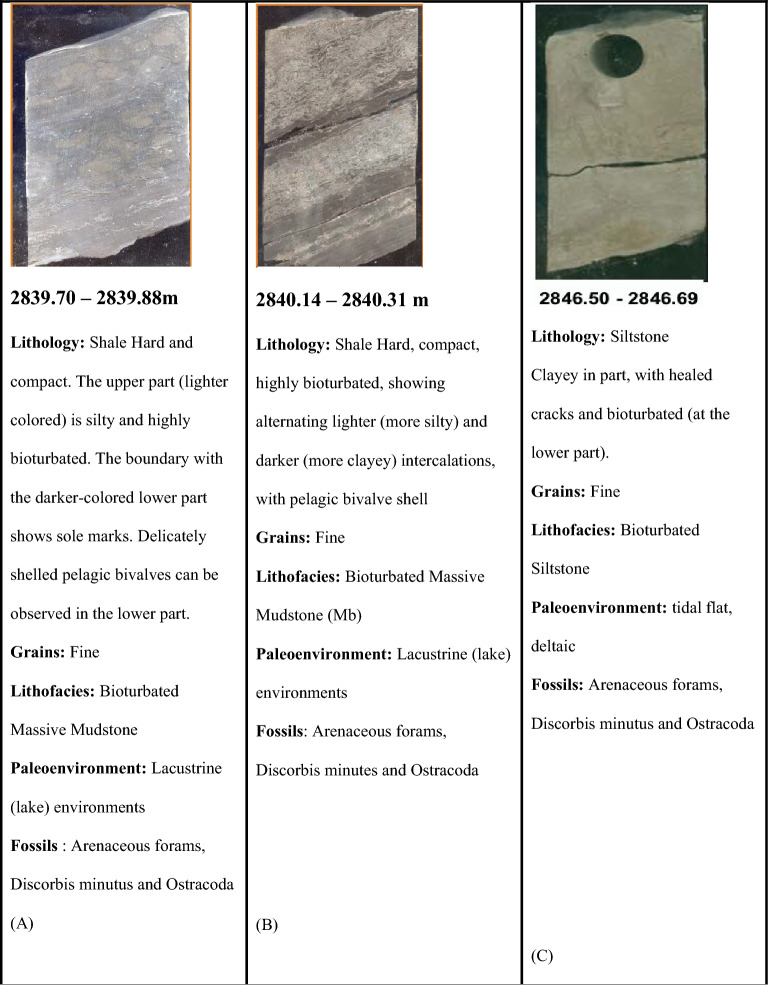

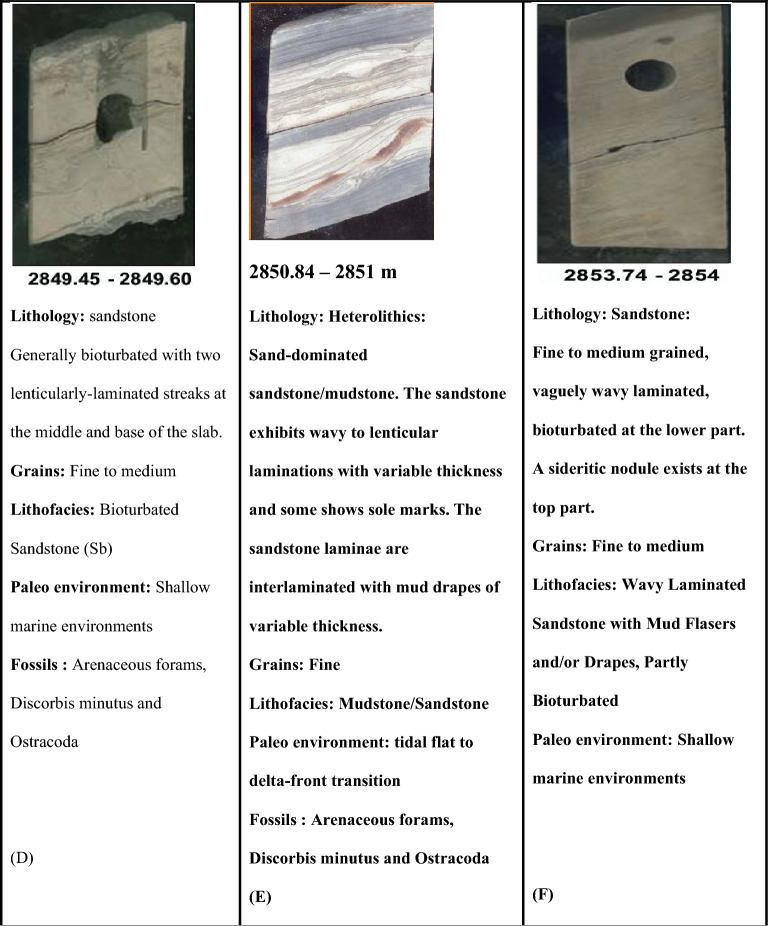

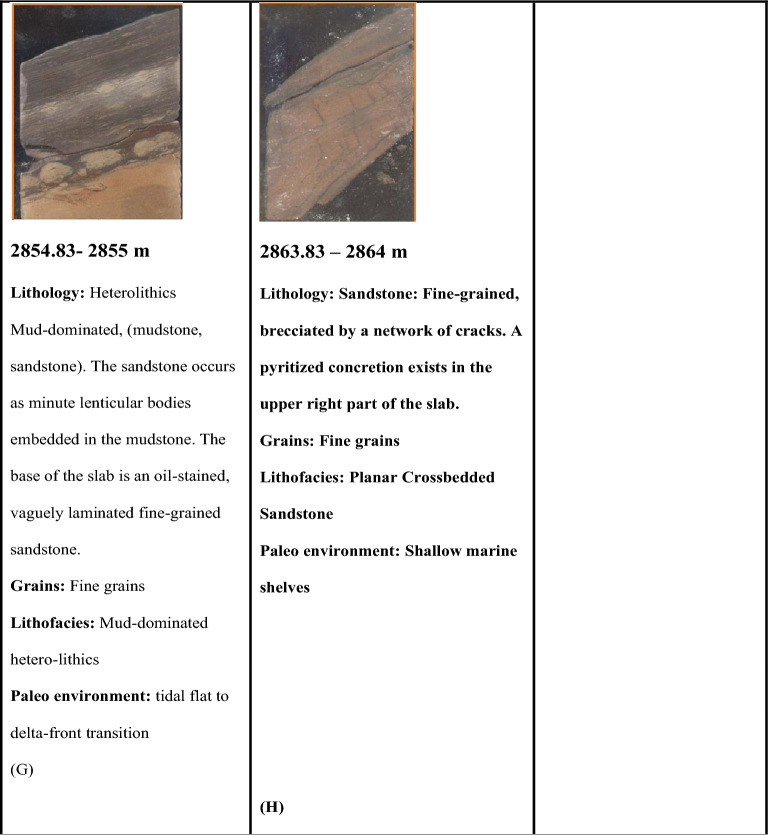
Different core slab intervals (**A**–**H**) in Abu Roash C Turonian age through Sitra8-17 well.

Slabbed conventional Core in Sitra 8–17 well between measured depths of 2837.20 -2864.21m. The dominant lithology is shale and sandstone, sandstone intervals likely act as good reservoirs, while shale or mudstone intervals act as seals. The Lithological heterogeneity, such as changes from clean sandstone to shale-rich intervals, can create heterogeneous flow units, influencing reservoir management^22,41,42^. The depositional environment is a tidally dominated deltaic depositional setting. Evidence for this is the Presence of bedded Coal (suggestive of the swamp) − Detection of herringbone cross lamination and recurrent occurrence of mud drapes, reactivation surfaces (suggestive of tidal influences) − Variable degrees of bioturbation − Presence of pelagic bivalves (marine conditions). − Common coarsening upward pattern for the sand bars (Badr El-Din repoer, 2024 unpublished). Figure 8 shows the detailed description of the lithology, sedimentary structure and lithofacies classification of ten slab core samples in the Late Cretaceous Abu Roash C Member.

The Abu Roash C in Sitra-8–17 well is composed of interbedded sandstone, shale, and carbonate layers, with moderate to poor reservoir quality in some intervals. Facies analysis suggests tidal flats, lagoonal systems, and shoreface deposits, indicating a low-energy, restricted marine environment^26,40^.

#### Petrophysical analysis

**Fig. 9:**
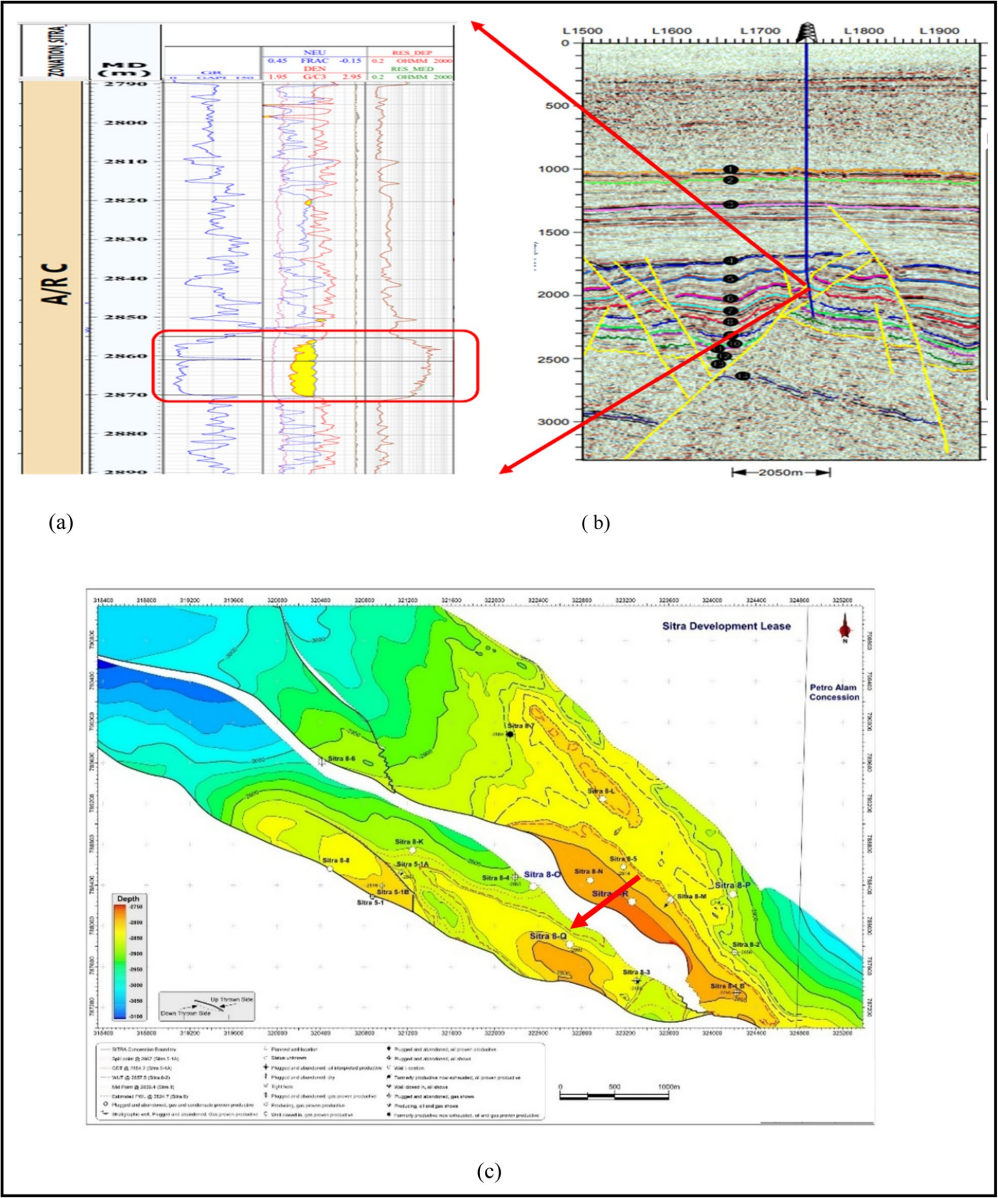
(**a**) Litho-saturation model showing petrophysical characteristics with highlighting zone of interest in Abu Roash C Member in Sitra8-17 (**b**) 2D interpreted seismic line through well SITRA8-17 (blue picked line represents top of Abu Roash C) (**c**) depth structure map.

Open hole log tools^21,43^, (Gamma-ray, Density, Neutron, and resistivity) reveal that the interval (2855-2864m) represents a promising reservoir quality, Fig. 9a with average porosity 13%, shale volume 9% and 59% water saturation.

#### Borehole image

**Fig. 10:**
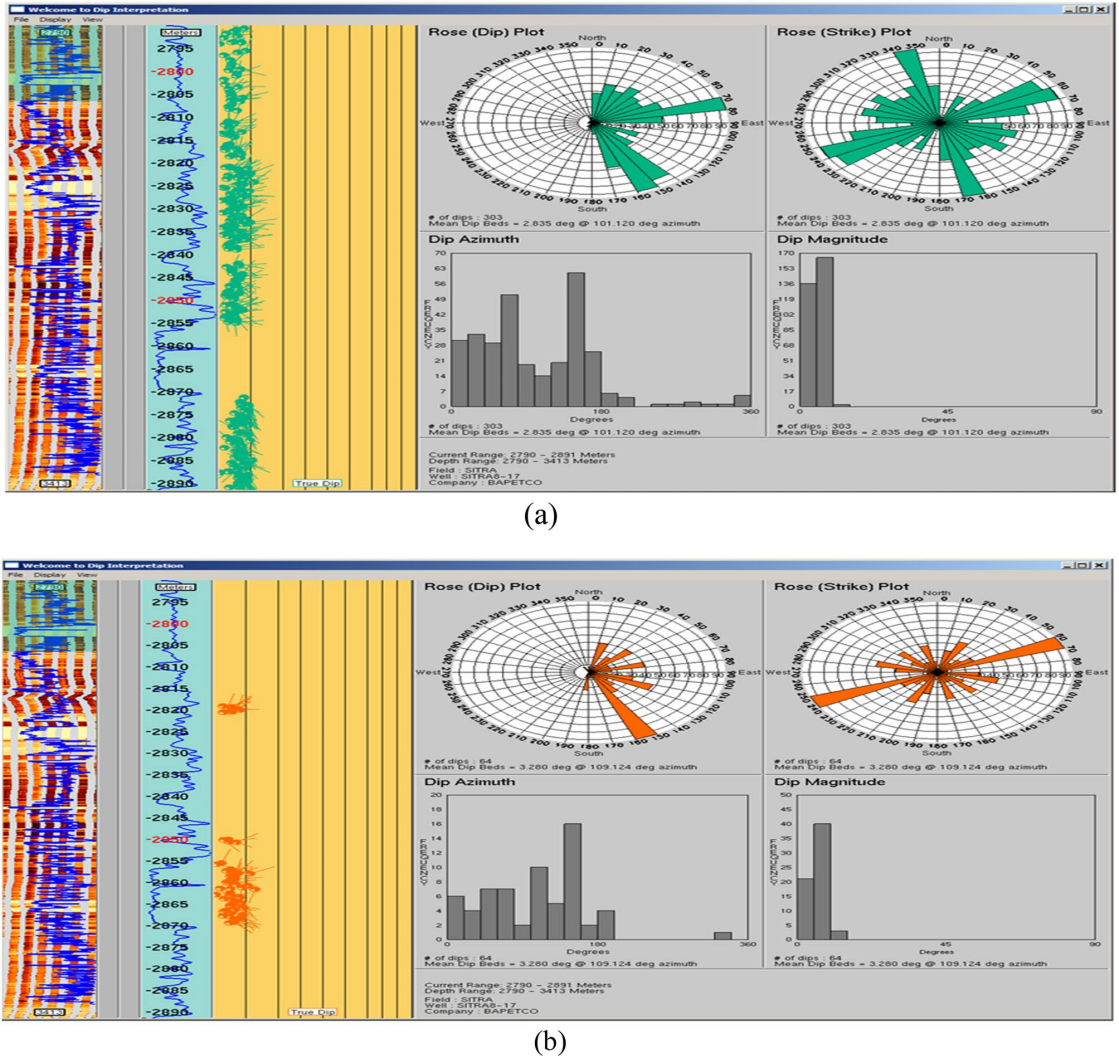
Rose plots and histograms for (**a**) the mudstone bed boundaries and lamination planes. They have two strike directions NE-SW & NW–SE and dip mostly due NE & SE with a calculated mean dip attitude measuring 2.8°/S80°E. And (**b**) few sandstone bed boundaries. They strike generally ENE-WSW and dip mostly due SSE with a calculated means dip attitude measuring 3.2°/S71°E. Picked from Abu Roash C Member.

**Fig. 11:**
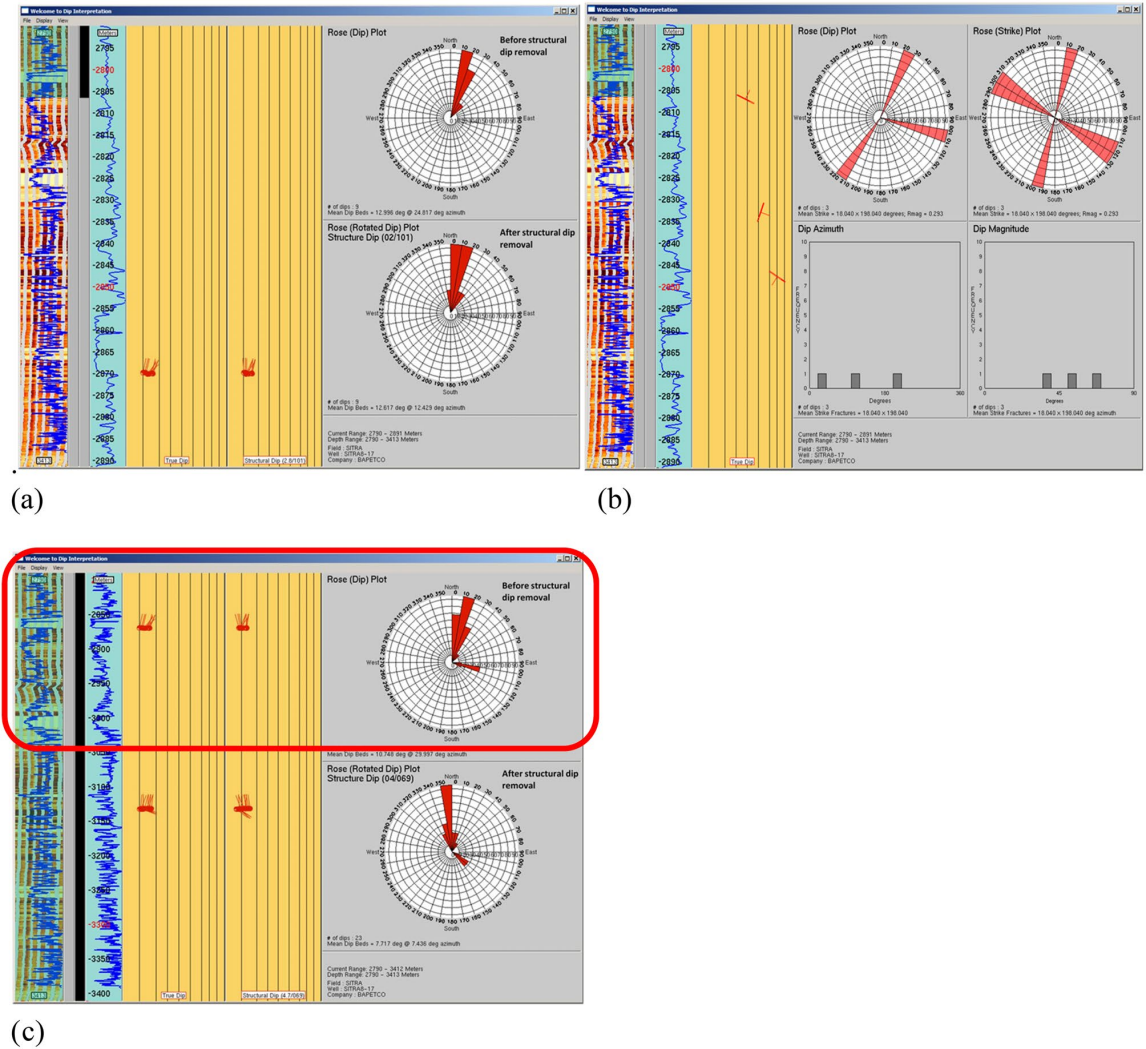
(**a**) Rose plots (before and after structural dip removal) for the cross-bedded sandstone intervals picked from the imaged section of Abu Roash C Member. (**b**) Rose plots and histograms presenting the attitudes of the 3 fractures cutting the Abu Roash C Member. (**c**) Rose plots (before and after structural dip removal) for the picked cross-laminated sandstone recorded in Abu Roash C. The cross beds dip generally due N indicating the direction of paleo-flow.

SITRA 8–17 well deviates with a maximum deviation of 14.71 deg at a depth of 2723m with borehole conditions generally good all over the logged interval without a significant rugosity, however, data in some intervals are partially affected by washouts and overpulls. In Sitra8-17 well, The Abu Roash C Member was drilled through the OMRI tool in the measured depth interval from 2790 m down to 2891 m with a penetrated thickness of about 101 m. The studied interval of this Member consists mainly of mudstone interbedded with limestone Fig. 10a and sandstone intervals Fig. 10b sandstone beds recorded in this unit mostly dip NNE direction and corrected mean dip attitude measuring 12.6°/N12°E. In Fig. 11a Three fractures were recognized in the imaged interval of Abu Roash C Member; two of them strike WNW-ESE and dips due to NNE and the third one is striking NNE-SSW and dips due to ENE with dip angles ranging from 35° to 65° Fig. 11b.

Cross-beds in sands are inclined planes being formed via a migration of straight crested sand waves or curved crested dunes, which generate and migrate either by stream water and wind currents or by wave and storm-related oscillation currents. Sand is transported up the wind /stream-ward (stoss) side of the bedform and is then transported by avalanche or grain flow down the steeper sheltered (leeward) side. Sand is deposited on the leeward side and accretes laterally resulting in progradation of the bedform and the development of inclined bedding (cross-bedding). The current flow direction is represented by the dip azimuth of the cross-forests. If tectonic deformation occurs during and after burial, then direct measurement of the cross-bed orientation in the rock record will not represent the original flow direction. To determine the depositional cross-bedding dip orientation and paleo-current flow direction, the structural (tectonic) dip needs to be determined and subtracted. Sitra 8–17 well, the mean structural dip attitude has been defined and the attitudes of cross-bedded sandstone sets recorded in Abu Roash C Member were corrected in Fig. 11c. The corrected cross-bedded sandstone indicates that the general paleo-flow that transported and accumulated those sands are generally toward North during Abu Roash C Member time.

**Fig. 12:**
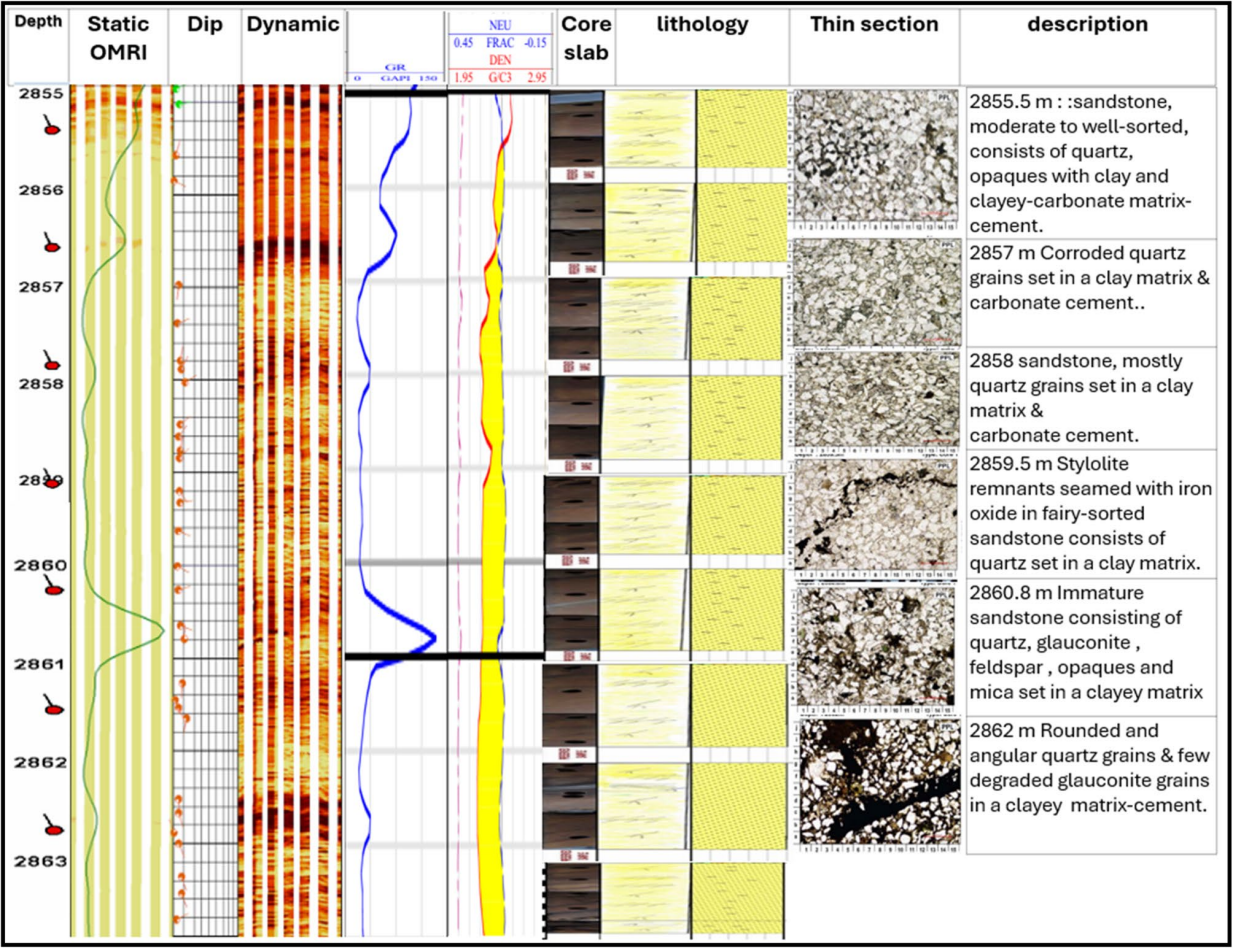
Integrating analysis of (borehole image, core and log data) in Abu Roash C Member well sitra8-17.

The integrated analysis of (borehole image, core and log data) in Abu Roash C Member in sitra8-17 well showed in Fig. 12, and they confirmed each other. The borehole image indicates that the interval between 2855 and 2864 m comprises sandstone, exhibiting low gamma-ray readings and a density-neutron crossover, which aligns with the sandstone and quartz identified in core samples from the same depth. These findings align with those reported in^22^, stating that Sitra 8–17 has two types of deposition: a clear coarsening upward pattern represented by the deposition of the mouth bars at the lower part, and a fining upward pattern for the tidal channel above the coal marker.

#### Seismic interpretation

The Sitra 8 Structure is a tilted fault block with several reservoirs of Upper Cretaceous Age at a depth ranging from 2700 to 3500 m. Oil-bearing reservoirs are encountered in the Abu Roash C, owing to extensional stresses, a NE-SW trending fault was formed where Sitra 8- 17 is drilled in the downthrown side. Figure 9b,c fault plane may act as a structural trap for Hydrocarbon accumulation^22,44^.

### Sitra8-3 well

**Fig. 13:**
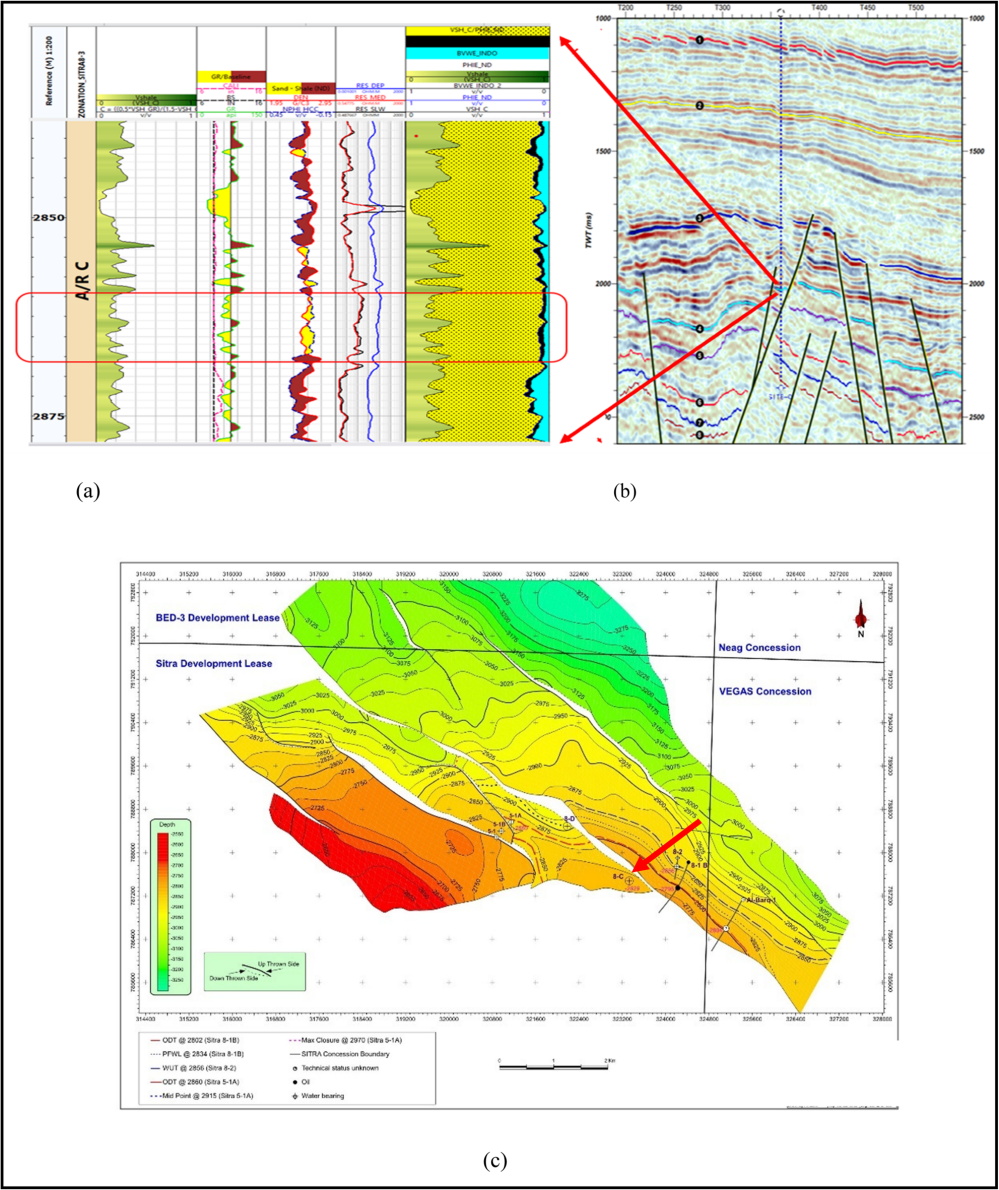
(**a**) characteristics with highlighting zone of interest in Abu Roash C Member in Sitra8-03 (**b**) seismic line through SITRA8-03 well (blue picked line represents top of Abu Roash C) (**c**) depth structure map.

Figure 13a shows the petrophysical analysis applied in SITRA8-03 well with highlighting interval (2860–2867)m, which reflects the responses of good reservoir quality characterized by 21% volume of shale, 9% porosity and 70% water saturation^43^. The seismic line in Fig. 13b,c shows that well Sitra8-03 was drilled in the upthrown side of a NE-SW normal fault; the upthrown side of normal faults can act as a structural trap for hydrocarbons. Hydrocarbon traps are more commonly associated with the footwall of the fault due to fault closure.

In alignment with^22,44^, the Abu Roash C Member consists predominantly of sandstone, shale, and siltstone. In the Sitra Field petroleum system, the primary source rock is widely interpreted to be the Khatatba Formation, while the Abu Roash C and G Members are key reservoir intervals. Hydrocarbon leakage in some wells has been attributed to poor fault seal behavior where G and C reservoirs are juxtaposed without effective sealing lithologies. In contrast, where mudstones or carbonates from either member are juxtaposed against sandstones, the fault may act as a seal, enhancing trap integrity^22,26,44^.

### Sitra8-15 well

**Fig. 14:**
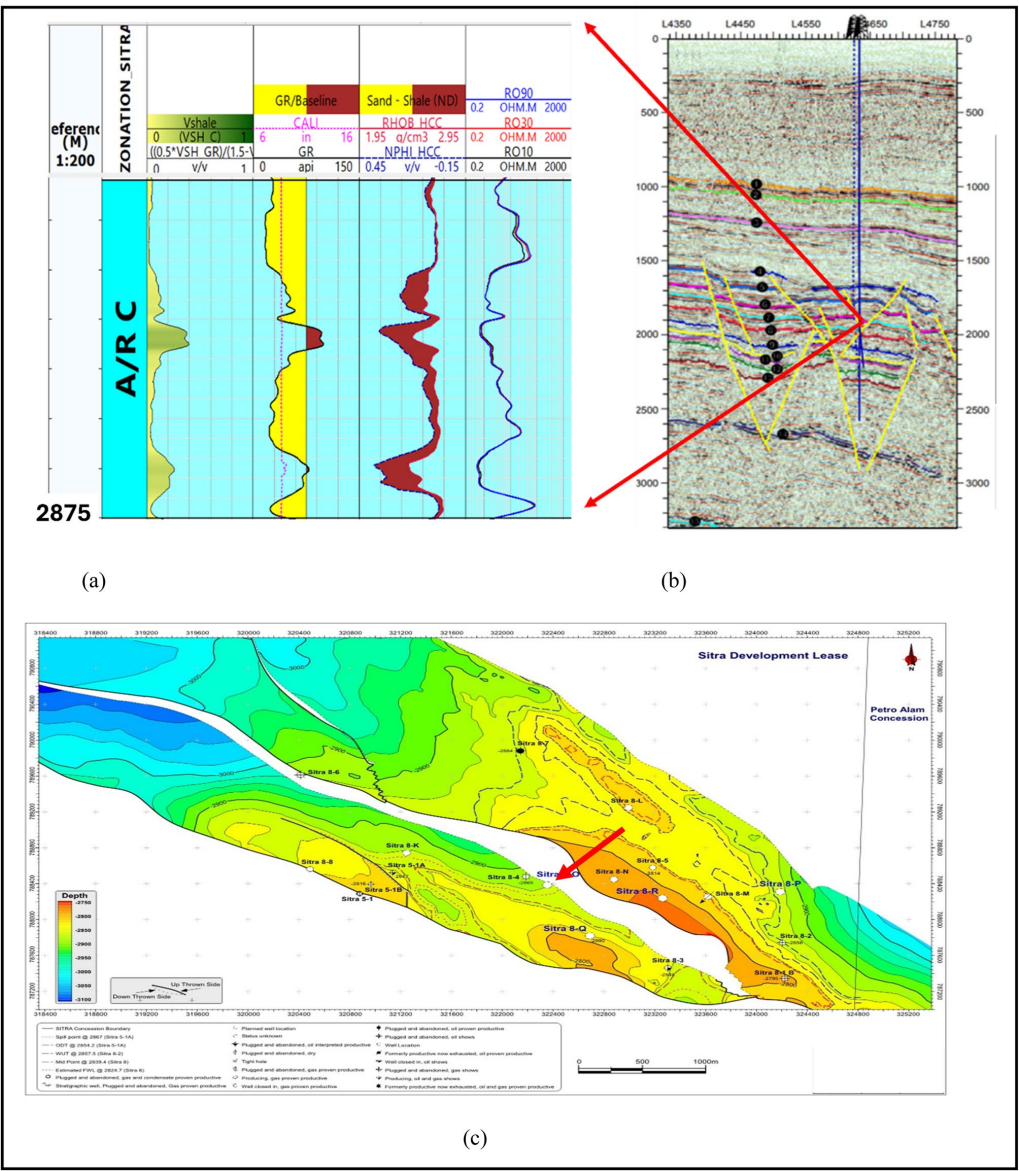
(**a**) characteristics with highlighting zone of interest in Abu Roash C Member in Sitra 8–15 well (**b**) seismic line through SITRA8-15 well (blue picked line represents top of Abu Roash C) (**c**) depth structure map.

From The results of the petrophysical analysis, Fig. 14a and the seismic line Fig. 14b,c. A thinning in the Abu Roash C Member layer was observed as the petrophysical analysis revealed that reservoir characteristics are exceedingly poor and the seismic lines indicated that the well was drilled in a narrow graben where the distance between the two faults is relatively small. A narrow graben can significantly affect the surrounding geological layers as the faults can displace layers, leading to mismatches in stratigraphy across the fault.

## Lithological correlation

**Fig. 15:**
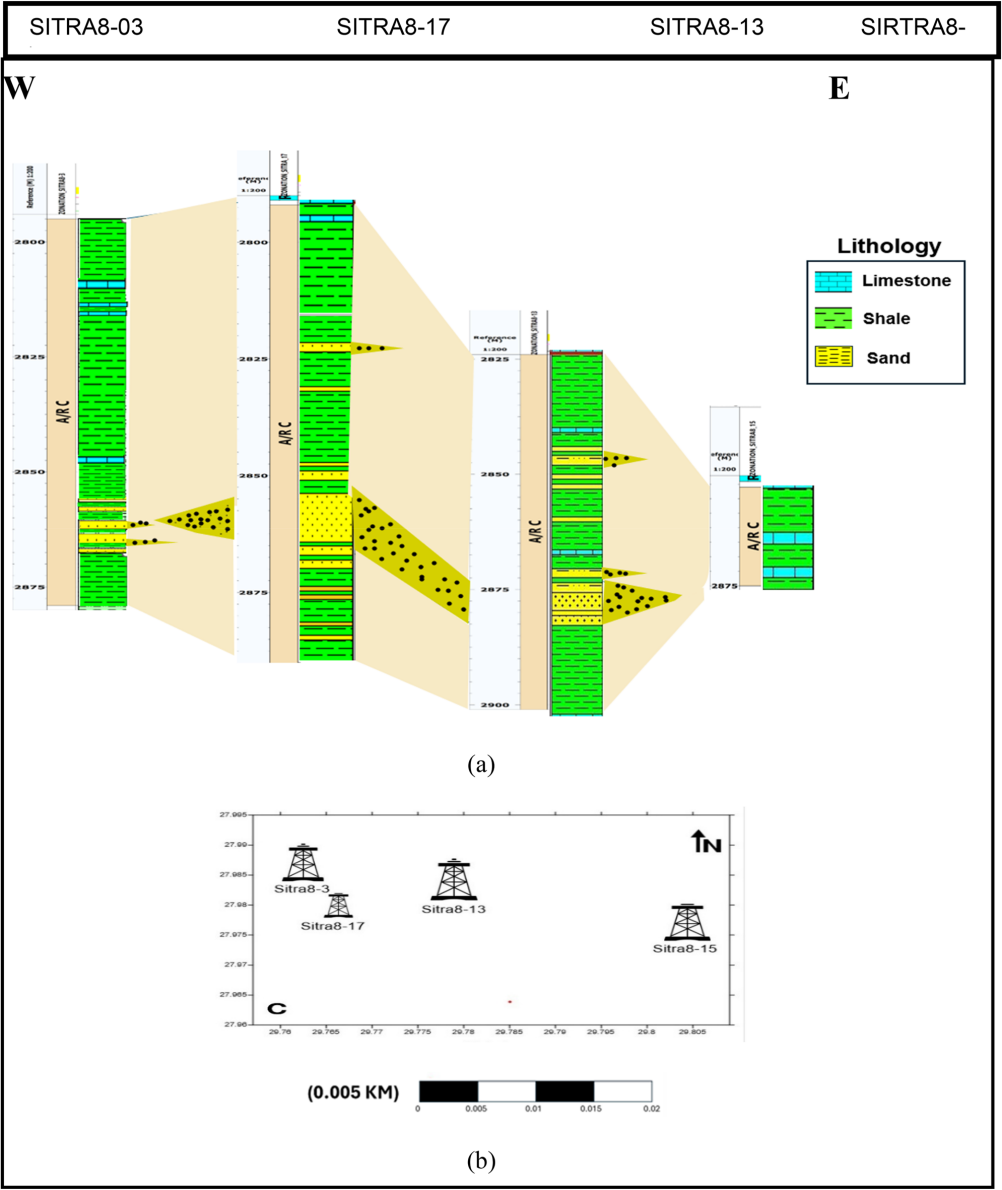
(**a**) Well correlation displaying the lithological variations of Abu Roash C across the four wells (**b**) Location map.

From the lithologic correlation Fig. 15, we can observe a thinning in Abu Roash C Member towards the east direction due to the erosion occured in the area, which contains a series of faults. Additionally, thin sandstone beds were noticed in well sitra8-03, and another sandstone bed was found in sitra8-17 well. This sandstone layer extended until well sitra8-13, where it tailed off in well sitra8-15. Abu Roash C Member in well Sitra8-15 is very thin and composed mainly of impermeable rock, which might act as a seal cap rock unit for the adjacent reservoirs.

## Results

The Analysis results show a high probability of hydrocarbons in both wells Sitra8-13 and Sitra8-17 (Table 4). Figure 15 demonstrates distinct sand intervals with favorable reservoir features based on the petrophysical investigation, and it confirms the data analysis we applied on wells.

**Table 4:** Summary of Analysis applied on Abu Roash C Member.

Well Name	Core analysis	Petrophysical analysis	Borehole image	Siesmic interpreatation
Well Sitra8-13	Upward-coarsening pattern	A dependable indicator of hydrocarbon presence	Sandstone and mudstone bed boundaries	Structural trap (Horst)
Well Sitra8-17	Coarsening upward pattern for the sand bars	Promising reservoir quality	Sandstone and Mudstone	Structural trap (Normal fault)
Well Sitra8-3	N/A	Good reservoir quality	N/A	Structural trap (Normal fault)
Well Sitra8-15	N/A	Poor reservoir quality	N/A	Narrow graben

## Conclusion

This study employed an integrated approach using borehole log data, seismic interpretation, and core analysis to assess the hydrocarbon prospectivity of the Abu Roash “C” Member in the Sitra-8 Field. The key findings are as follows:

1. Depositional Setting: The Abu Roash “C” Member exhibits features of a tidally influenced deltaic system, reinforcing its role as a reservoir-bearing unit in the Sitra area.
2. Paleoenvironment : A shallow marine to marginal marine setting, likely influenced by tidal and storm processes
3. Reservoir Quality – Sitra-8–13: The interval from 2873 to 2880 m displays favorable reservoir attributes, including cross-bedded sandstone with low gamma-ray readings, well-connected microporosity, and an average effective porosity of approximately 12%.
4. Reservoir Quality – Sitra-8–17: The 2855–2864 m zone shows strong alignment between core, image, and log data, with an average porosity of 13.4%, indicating high-quality reservoir potential.
5. Reservoir Quality – Sitra-8–03: A thinner sand body between 2860 and 2867 m reveals good reservoir characteristics, albeit more restricted in lateral extent.
6. Non-Reservoir Zone – Sitra-8–15: This well lacks significant reservoir development, with a notably thinner Abu Roash “C” interval compared to surrounding wells.
7. Structural Traps: Seismic analysis identifies NW–SE oriented fault systems compartmentalizing the Abu Roash “C” closure, which enhances the formation of discrete structural traps.
8. Seal Capacity: Beyond its reservoir potential, the Abu Roash “C” Member may act as an effective regional seal, given its impermeable lithology—though sealing efficiency is highly dependent on the local structural configuration.
9. Facies Variability: Correlation across wells shows eastward thinning of the Abu Roash “C” Member, with high-quality sands observed in Sitra-8–13 and Sitra-8–17 pinching out toward Sitra-8–15. A thin sand interval is also noted in Sitra-8–03.

Taken together, these results affirm the dual role of the Abu Roash “C” Member as both a promising hydrocarbon reservoir and a potential regional seal, shaped by local depositional dynamics and structural complexity.

## Data Availability

The data supporting the findings of this study are provided by Badr El-Din company. However, due to licensing re-strictions, these data are not publicly accessible. They can be obtained from the corresponding author upon reasonable request and with prior approval from the Egyptian General Petroleum Corporation.

## References

[1] Abdel-Fattah, M. I., Sarhan, M. A., Ali, A. S. & Hamdan, H. A. Structural and petrophysical characteristics of the Turonian “AR/G ″reservoirs in heba field (Western Desert, Egypt): Integrated approach for hydrocarbon exploration and development. *J. Afr. Earth Sci.* **207**, 105072 (2023).

[2] Pavlovic, M., and Markovic, M., A New Approach for Interpreting Lithofacies and Sequence Stratigraphy using Borehole Image Data in Wells Drilled with Non-Conductive Mud Systems. Annual Conference & Exhibition, Salt Lake City, Utah. (2003).

[3] Sarhan, M. A. & Abdel-Fattah, M. I. Geophysical evaluation and petrophysical assessment of the Abu Roash “F” Member: A probable unconventional oil reservoir in Heba Field, eastern Abu El-Gharadig Basin, Egypt. *J. African Earth Sci.* **217**, 105330 (2024).

[4] Sarhan, M. A. & Selim, E. S. Geophysical appraisal of fractured carbonate reservoirs: a case study of Abu Rash D member, Abu El-Gharadig field, western desert, Egypt. *Euro Mediterranean J. Environ. Integr.* **8**(2), 395–408 (2023).

[5] Taha, M., Abdelhafeez, T., El-hady, S., Nooh, A. & Mohamed, W. Improving hydrocarbon prospect evaluation at Badr El Din-3 and Badr El Din-15 oil fields by integrating 3D structural modeling and petrophysical analysis for Abu-Gharadig Basin, North of Western Desert, Egypt. *Arab. J. Sci. Eng.* **48**, 625–643 (2022).

[6] Catuneanu, O. Sequence stratigraphy: Guidelines for a standard methodology. *Stratigr. Timescales* **2**, 1–57 (2017).

[7] Abdelwahed, A., Gamal, M. & Metwally, A. Mapping the subsurface structures and contacts using aeromagnetic data (Structural and petrophysical: central and northern Egypt). *Iran. J. Geophys.* **17**(3), 13–25 (2023).

[8] Abuzaied, M., Mabrouk, W., Metwally, A., Bakr, A. & Sharaf Eldin, Sh. Correlation of the reservoir characteristics from the well-logging data and core measurements in QASR field, north Western Desert, Egypt. *Arab. J. Geosci.* **1**(13), 118 (2020).

[9] Abuzaied, M., Metwally, A., Mabrouk, W., Khalil, M. & Bakr, A. Seismic interpretation for the Jurassic/Paleozoic reservoirs of QASR gas field, Shushan-Matrouh Basin north Western Desert, Egypt. *Egypt. J. Pet.* **1**(28), 103–110 (2019).

[10] Suez Amer, M., Mabrouk, W. M. & Eid, A. M. Petrophysical assessment of the Hammam Faraun, Matulla and Nubia Reservoirs in the Ashrafi Oil Field, Gulf of A. *Sci. Rep.* **15**(1), 3326 (2025). 39865084 10.1038/s41598-025-86297-0 PMC11770147

[11] Chikiban, B., Kamel, M., Mabrouk, W. & Metwally, A. Petrophysical characterization and formation evaluation of sandstone reservoir: Case study from Shahd field, Western Desert, Egypt. *Contrib. Geophys. Geodesy* **52**, 443–466 (2022).

[12] Eid, A., Mabrouk, W., Amer, M. & Metwally, A. 3D structural modeling using seismic data and well logs for Khatatba reservoir in Matruh-Shushan Basin, North Western Desert, Egypt. *Sci. Rep.* **13**, 20158 (2023). 37978307 10.1038/s41598-023-47487-w PMC10656560

[13] Eid, A. M., Amer, M., Mabrouk, W. M., El-khteeb, A. & Metwally, A. Delving into the Jurassic sediments of the Matruh Basin, Northwestern Desert, Egypt: A multidisciplinary approach using seismic data, stratigraphic analysis, and 3D facies modeling. *Mar. Pet. Geol.* **177**, 107376 (2025).

[14] Eid, A., Mabrouk, W. & Metwally, A. Formation evaluation and petrophysical analysis of well logging data: an example from Matruh-Shushan Basin, North Western Desert, Egypt. *Iraqi J. Sci.* **65**(8), 4336–4358 (2023).

[15] Hassan, A., Mabrouk, W. & Farhoud, K. Petrophysical analysis for Ammonite-1 well, Farafra Area, Western Desert, Egypt. *Arab. J. Geosci.* **7**, 5107–5125 (2013).

[16] Mahmoud, A., Metwally, A., Mabrouk, W. & Leila, M. Controls on hydrocarbon accumulation in the pre-rift paleozoic and late syn-rift cretaceous sandstones in PTAH oil field, north Western Desert, Egypt: Insights from seismic stratigraphy, petrophysical rock-typing and organic geochemistry. *Mar. Pet. Geol.* **155**, 106398 (2023).

[17] Noureldin, A., Mabrouk, W. & Metwally, A. Delineating tidal channel feature using integrated post-stack seismic inversion and spectral decomposition applications of the Upper Cretaceous reservoir Abu Roash C: A case study from Abu-Sennan oil field, Western Desert, Egypt. *J. African Earth Sci.* **205**, 104974 (2023).

[18] Noureldin, A., Mabrouk, W. & Metwally, A. Structural and petroleum system analysis using seismic data interpretation techniques to Upper Cretaceous rock units: A case study, West Abu-Sennan oil field, Northern Western Desert, Egypt. *J. African Earth Sci.* **198**, 104772 (2023).

[19] Noureldin, A., Mabrouk, W. & Metwally, A. Superimposed structure in the southern periphery of Abu El-Gharadig Basin, Egypt: Implication to petroleum system. *Contrib. Geophys. Geodesy* **53**(2), 97–110 (2023).

[20] Noureldin, A., Mabrouk, W., Chikiban, B. & Metwally, A. Formation evaluation utilizing a new petrophysical automation tool and subsurface mapping of the Upper Cretaceous carbonate reservoirs, the southern periphery of the Abu-Gharadig Basin, Western Desert, Egypt. *J. African Earth Sci.* **205**, 104977 (2023).

[21] Mohamed, H., Mabrouk, W. & Metwally, A. Delineation of the reservoir petrophysical parameters from well logs validated by the core samples case study Sitra field, Western Desert, Egypt. *Sci. Rep.* **14**, 26841 (2024). 39500975 10.1038/s41598-024-77371-0 PMC11538348

[22] Salama, H., Darwish, M., Wahdan, M. & El-Batal, A. Identifying redevelopment concepts to enhance Abu Roash” oil reservoir productivity Sitra Area, Abu El-Gharadig Basin, Western Desert, Egypt. *Egypt. J. Pet.* **26**(2), 235–267 (2017).

[23] Bdr El-Din Company core sample Report, (2024).

[24] Sarhan, M. Geophysical appraisal for the sandy levels within Abu Roash C and E members in Abu El-Gharadig Field, Western Desert, Egypt. *J. Pet. Explor. Prod. Technol.* **11**, 1101–1122 (2021).

[25] Assal, E., Selim, El. & Hedaihed, S. Facies analysis, depositional architecture, and sequence stratigraphy of the upper Abu Roash “G” Member (Late Cenomanian), Sitra Field, Western Desert, Egypt. *Arab. J. Geosci.* **14**, 1150 (2021).

[26] El-Kharboutly, K., Helal, A. & Aly, S. 3D Seismic interpretation and structural analysis of Sitra-8 field in Abu El-Gharadig Basin, Northern Western Desert, Egypt. *Egypt. J. Geol.* **64**, 33 (2020).

[27] Gharieb, A., Adel, M., Algarhy, A., Farid, A. & Darraj, N. *Optimizing development strategies for unconventional reservoirs of Abu Roash Formation in Western Desert of Egypt* (Society Exploration Geophysicists, 2024).

[28] Farouk, S. et al. Assessment of the Upper Cretaceous Abu Roash carbonate source rocks from the Beni Suef field, Western Desert, Egypt. *J. African Earth Sci.* **215**, 105272 (2024).

[29] Mostafa, T. et al. Exploring hydrocarbon potential with 3D modeling techniques: Lower Cretaceous formations in Abu Sennan field, north Western Desert. *Adv. Res. Evolv. Sci.* **11**, 158–173 (2025).

[30] Schlumberger, Well Evaluation Conference, Egypt. (1995).

[31] Hameedy, E. L. Detection of karst features and associated geohazard using ground penetrating radar and 2D electrical resistivity imaging; case study from Sannur protectorate, Egypt. *Contrib. Geophys. Geodesy* **53**(3), 167–190 (2023).

[32] Osman, M., Ahmed, H. & Hosny, A. A comparative petrophysical evaluation of the Abu Roash, Bahariya, and Kharita reservoirs using well-logging data, East El-Fayoum, Egypt. *Sci. Rep.* **15**, 2732 (2025). 39837907 10.1038/s41598-024-83332-4 PMC11751110

[33] Elhossainy, M., Shafeiy, M., Al-Areeq, N. & Hamdy, D. Petroleum generation modeling of the Middle-Late Cretaceous sediments in the Abu El-Gharadig Field, Northwestern Desert, Egypt. *Geol. J.* **57**(9), 3851–3880 (2022).

[34] Moustafa, A. et al. Structural setting and tectonic evolution of the Bahariya Depression, Western Desert, Egypt. *Geo Arabia* **8**(1), 91–124 (2003).

[35] Metwalli, F. I. & Shendi, E. H. Core and well logs interpretation for better reservoir characterization in Shushan Basin, Egypt. *Arab. J. Geosci.* **14**, 2587. 10.1007/s12517-021-08819-0 (2021).

[36] Metwalli, F. I. & Shendi, E. H. Seismic facies analysis of thin sandstone reservoirs, North Western Desert, Egypt. *J. Pet. Explor. Prod. Technol.* **9**, 793–808. 10.1007/s13202-018-0541-5 (2019).

[37] Eid, A. M., Mabrouk, W. M., Amer, M. & Metwally, A. Reservoir modeling of heterogeneities, structures, and petrophysical properties of the Berenice Oil Field: Implications for sustainable management and CO2 storage in the North Western Desert, Egypt. *Mar. Pet. Geol.* **180**, 107466 (2025).

[38] Li, B. et al. Introduction to special section: Borehole image data applications in reservoir characterization—Case studies and updates on new developments. *Soc. Explor. Geophysicists Am. Assoc. Petroleum Geol.* **12**(2), 1 (2024).

[39] Sanderson, D. J. & Peacock, D. C. P. Making rose diagrams fit-for-purpose. *Earth Sci. Rev.* **201**, 103055 (2020).

[40] Fagelnour, M. S. & Shendi, M. F. I. Structural and facies modeling of the Lower Cretaceous Alam El Bueib reservoirs in the Shushan Basin, Western Desert, Egypt. *Arab. J. Geosci.* **11**(9), 1–24 (2018).

[41] Salem, I., Ghazala, H. & El-Diasty, W. Prospect evaluation of BED 3 and Sitra oil fields, Abu El-Gharadig Basin, North Western Desert, Egypt. *Natl. Res. Inst. Astron. Geophys.* **4**, 222–235 (2019).

[42] Shalaby, A. & Sarhan, M. A. Origin of two different deformation styles via active folding mechanisms of inverted Abu El Gharadiq Basin, Western Desert, Egypt. *J. African Earth Sci.* **183**, 104331 (2021).

[43] Amer, M., Mabrouk, W., Soliman, K., Eid, A. & Metwally, A. Formation evaluation of middle Miocene reservoirs via petrophysical analysis of well logging data; case study from Southern part of Gulf of Suez, Egypt. *Iran. J. Geophys.* **18**(3), 39–57 (2024).

[44] Barakat, M. & Nooh, A. Reservoir quality using the routine core analysis data of Abu Roash “C” in Badr El Din-15 oil field, Abu El-Gharadig Basin, northwestern Desert, Egypt. *J. African Earth Sci.* **129**, 683–691 (2017).

